# Regulation of cardiomyocyte behavior in zebrafish trabeculation by Neuregulin 2a signaling

**DOI:** 10.1038/ncomms15281

**Published:** 2017-05-09

**Authors:** S. Javad Rasouli, Didier Y. R. Stainier

**Affiliations:** 1 Department of Developmental Genetics, Max Planck Institute for Heart and Lung Research, 61231 Bad Nauheim, Germany

## Abstract

Trabeculation is crucial for cardiac muscle growth in vertebrates. This process requires the Erbb2/4 ligand Neuregulin (Nrg), secreted by the endocardium, as well as blood flow/cardiac contractility. Here, we address two fundamental, yet unresolved, questions about cardiac trabeculation: why does it initially occur in the ventricle and not the atrium, and how is it modulated by blood flow/contractility. Using loss-of-function approaches, we first show that zebrafish Nrg2a is required for trabeculation, and using a protein-trap line, find that it is expressed in both cardiac chambers albeit with different spatiotemporal patterns. Through gain-of-function experiments, we show that atrial cardiomyocytes can also respond to Nrg2a signalling, suggesting that the cardiac jelly, which remains prominent in the atrium, represents a barrier to Erbb2/4 activation. Furthermore, we find that blood flow/contractility is required for Nrg2a expression, and that while non-contractile hearts fail to trabeculate, non-contractile cardiomyocytes are also competent to respond to Nrg2a/Erbb2 signalling.

## Abstract (web summary)

Cardiac trabeculae (which are sponge-like muscular structures) form mostly as a result of cardiomyocyte (CM) delamination in zebrafish. Here, the authors identify Nrg2a in zebrafish as a key regulator of trabeculation, and atrial and non-contractile CMs also respond to Nrg2a despite not forming trabeculae.

Cardiac trabeculation, which allows the increase in muscle mass prior to the formation of coronaries, is one of the most important processes the vertebrate heart must undergo to form and function properly^1^. During this process, trabeculae, which are sponge-like muscular structures in the cardiac cavity, form as a result of cardiomyocyte delamination and proliferation^2^,^3^,^4^,^5^,^6^. Defects in this complex morphogenetic event lead to embryonic lethality or adult onset dilated cardiomyopathy^5^,^7^,^8^,^9^,^10^,^11^,^12^ illustrating the importance of trabeculation in cardiac development and function.

The vertebrate heart initially consists of two cell layers, the endocardium and myocardium, separated by an acellular, gelatinous matrix called the cardiac jelly^13^,^14^,^15^. Ablating components of the cardiac jelly by injecting hyaluronidase in chick embryos has been reported to cause looping defects^16^, indicating that it plays an important role during early cardiac morphogenesis. Endocardial–myocardial interactions are known to orchestrate cardiac trabeculation; for example, in chick embryos, a myocardial-derived growth factor, Angiopoietin 1, has been reported to bind to its receptor Tie2 which is expressed by endocardial cells and modify their angiogenic behaviour which in turn affects the complexity of cardiac trabeculation^17^. Similarly, signalling from endocardial to myocardial cells is also necessary for trabeculation. For instance, mice lacking the endocardial-derived ligand Nrg1 or its receptors Erbb2 and Erbb4, which are expressed by myocardial cells, exhibit severe trabeculation defects^7^,^8^,^12^,^18^. Similarly, zebrafish *erbb2* mutants fail to form trabeculae^5^. Additional studies have shown that Nrg signalling can positively regulate cardiomyocyte proliferation and heart regeneration^19^,^20^,^21^,^22^.

Cardiac formation can also be influenced by its function^23^,^24^,^25^. Recent studies have shown the dependence of trabeculation on physical forces generated by cardiac contractility and blood flow^6^,^26^,^27^. For instance, several groups have reported that reduction of blood flow in the ventricular chamber markedly diminishes trabeculation in chick embryos and zebrafish larvae^6^,^26^,^28^,^29^. By manipulating cardiac contractility, another study presented data indicating that cardiac contraction promotes trabeculation through the regulation of Notch signalling in the ventricular endocardium^27^. These and other findings support the hypothesis that physical forces are necessary in the developing heart, particularly during trabecular formation.

In this study, we use the zebrafish model to address two fundamental questions pertinent to cardiac trabeculation; first, why trabeculae initially form in the ventricle and not the atrium, as is observed in zebrafish as well as in other vertebrates including amniotes^3^,^30^, and second, how trabeculation is modulated by physical forces. Using a genetic approach and high-resolution live imaging, we first show that Nrg2a is an essential ligand for cardiac trabeculation in zebrafish. By ectopically expressing *nrg2a* in cardiomyocytes, we further show that atrial cardiomyocytes, like ventricular cardiomyocytes, can respond to Nrg2a/Erbb2 signalling. Using loss- and gain-of-function approaches, we show that blood flow/contractility modulates endocardial *nrg2a* expression while it is not required for the expression or function of Nrg2a/Erbb2 signalling effectors in cardiomyocytes.

## Results

### Nrg2a is required for cardiac trabeculation in zebrafish

Given the role of Nrg1 in cardiac trabeculation in mouse^7^,^8^,^12^,^18^, we mutated zebrafish *nrg1* using TALEN technology^31^. Since *nrg1* has multiple isoforms^32^, we targeted exon 2 which encodes part of the highly conserved IGc2-domain, and identified a Δ14 allele (Supplementary Fig. 1a) encoding a predicted truncated protein (Supplementary Fig. 1b) due to harbouring a premature stop codon within the same exon. Using brightfield microscopy, no gross morphological defects were observed in *nrg1* Δ14 (*nrg1*^*bns101*^) homozygous mutant larvae (Supplementary Fig. 1c,d). Incrossing *nrg1*^*bns101/+*^ fish in the *Tg(myl7:LIFEACT-GFP)* background, a myocardial specific line in which F-actin (filamentous actin) is labelled with GFP^33^, we observed that trabeculation appears unaffected in *nrg1*^*bns101*^ mutant larvae (Supplementary Fig. 1e–h), which can survive to become fertile adults.

**Figure 1:**
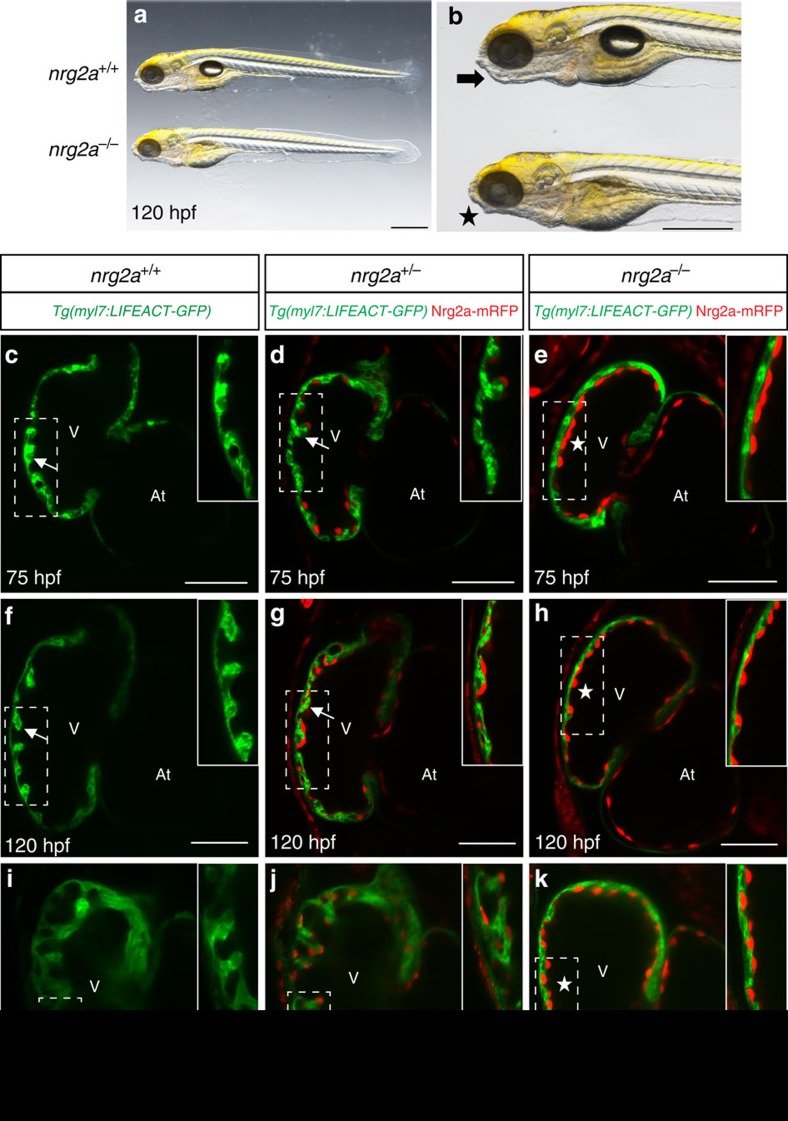
Nrg2a is required for cardiac trabeculation in zebrafish. (**a**,**b**) Zebrafish larvae from *nrg2a*^*+/−*^ incrosses were imaged at 120 hpf; lateral view, anterior to the left; scale bars, 0.5 mm. *nrg2a*^*−/−*^ larvae can be recognized by their defective jaws (asterisk in **b**). (**c**–**k**) *nrg2a* mutants lack cardiac trabeculae. Confocal images (mid-sagittal sections) of larval hearts from *Tg(myl7:LIFEACT-GFP);nrg2a*^*+/−*^ incrosses at 75 (**c**–**e**), 120 (**f**–**h**) and 168 hpf (**i**–**k**); ventricular outer curvature (dashed boxes) magnified in upper right corners; arrows and asterisks indicate trabeculated and non-trabeculated walls, respectively; scale bars, 50 μm.

Next, we decided to investigate the role of Nrg2a, another member of the Nrg family, by using a conditional protein-trap line, *nrg2a*^*mn0237Gt*^ (refs 34, 35). Using brightfield microscopy, *nrg2a* mutants can be recognized by their aberrant jaws (Fig. 1a,b) as well as other defects in median fin fold (MFF) morphogenesis and swim bladder inflation^35^. As previously reported, the gene-breaking transgene (GBT) *nrg2a* allele generates a truncated Nrg2a protein tagged with mRFP (Nrg2a-mRFP) due to the in-frame integration of the mutagenic pGBT-RP2.1 (RP2) cassette in the intron between alternative exon 1C and exon 2 (ref. 35). Prior studies have also shown that the expression of Nrg2a-mRFP recapitulates that of endogenous *nrg2a* (refs 34, 35). Confocal imaging of larvae from *nrg2a*^*+/−*^ incrosses in the *Tg(myl7:LIFEACT-GFP)* background (Fig. 1c–k) revealed that Nrg2a-mRFP is highly expressed in the endocardium at early larval stages (Fig. 1d,e,g,h,j,k), and that *nrg2a*^*−/−*^ hearts fail to form trabeculae by 75 h post fertilization (hpf) (Fig. 1c–e). Observation of *nrg2a* mutants at later stages, including 120 and 168 hpf (Fig. 1f–k), confirmed that their trabeculation defects appear as severe as those in *erbb2* mutants^5^. Overall, these data suggest that Nrg2a, and not Nrg1, is the crucial regulator of cardiac trabeculation in zebrafish.

### Differential Nrg2a expression in the ventricle and atrium

**Figure 2:**
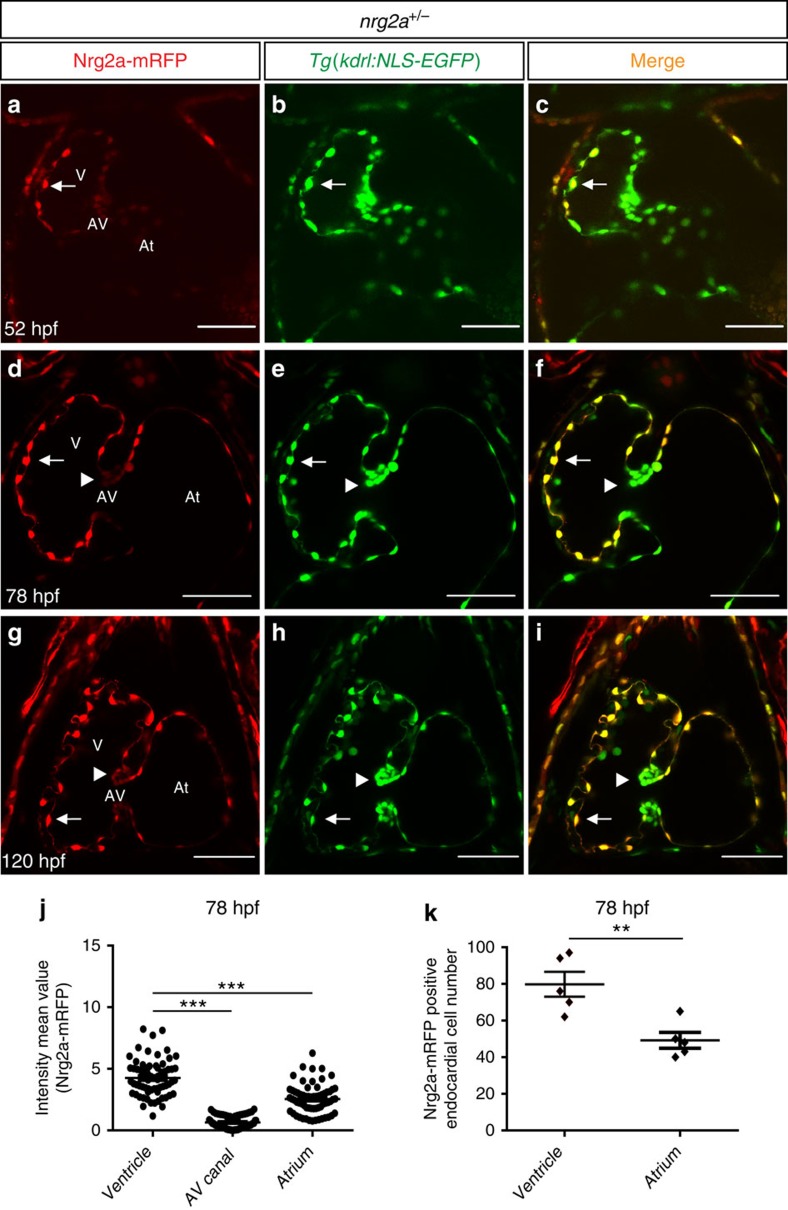
Nrg2a-mRFP expression during embryonic and larval cardiac development. (**a**–**i**) Two-dimensional (2D) confocal images (mid-sagittal sections) of zebrafish hearts from *Tg(kdrl:NLS-EGFP);nrg2a*^*+/−*^ outcrosses at 52 (**a**–**c**), 78 (**d**–**f**) and 120 hpf (**g**–**i**) showing that Nrg2a-mRFP expression is clearly visible in the ventricular endocardium by 52 hpf, mainly in the outer curvature (**a**–**c**), and that it becomes stronger in both ventricular and atrial chambers at 78 and 120 hpf (**d**–**i**); however, it is weak in the atrioventricular canal; arrowheads point to the superior valve leaflet (**a**–**i**); AV, atrioventricular canal, At, atrium; V, ventricle; scale bars, 50 μm. (**j**) Cell-based mRFP intensity, measured with the ZEN Imaging Software and plotted as a graph, showing that Nrg2a-mRFP is more highly expressed in the ventricle compared to the AV canal and atrium; dots in this graph represent individual Nrg2a-mRFP expressing endocardial cells. (**k**) Nrg2a-mRFP positive endocardial cells counted in each chamber at 78 hpf, showing that there are more Nrg2a-mRFP positive endocardial cells in the ventricle than in the atrium at 78 hpf; dots in this graph represent individual hearts; *N*=5 hearts; values represent means±s.e.m.; **P≤0.01, ***P≤0.001 by Student's *t*-test.

**Figure 3:**
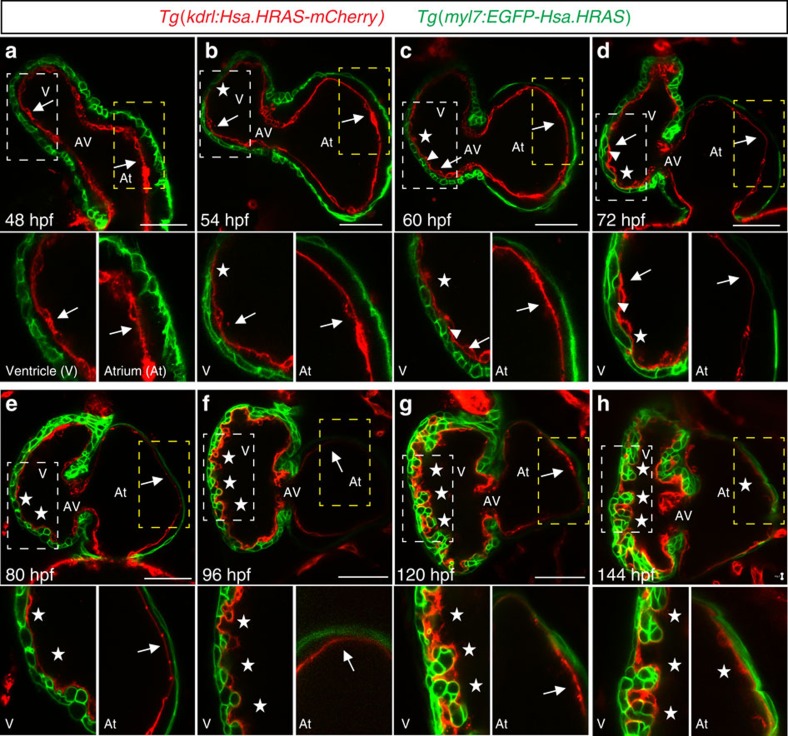
Reduction of cardiac jelly thickness in developing zebrafish. (**a**–**h**) Mid-sagittal confocal sections of late embryonic and early larval zebrafish hearts. Animals from *Tg(kdrl:Hsa.HRAS-mCherry);Tg(myl7:EGFP-Hsa.HRAS)* incrosses were synchronized at the tail bud stage (10 hpf) and imaged at 48 (**a**), 54 (**b**), 60 (**c**), 72 (**d**), 80 (**e**), 96 (**f**), 120 (**g**) and 144 hpf (**h**); endocardial and myocardial membranes are labelled in red and green, respectively; AV, atrioventricular canal, At, atrium; V, ventricle; scale bars, 50 μm. Higher magnification images of outer curvature area of ventricular (white dashed boxes) and atrial (yellow dashed boxes) walls are shown beneath each time point, respectively. The cardiac jelly is initially thicker in the atrium than in the ventricle at 48 hpf (**a**). It is greatly reduced in the ventricle by 72 hpf, (**b**–**d**) and almost fully gone by 96 hpf, while it is still detectable at 120 hpf in the atrium (**e**–**h**); arrowheads point to trabecular cardiomyocytes; arrows and asterisks indicate the presence and absence of cardiac jelly, respectively.

Trabeculae start to form in zebrafish around 60 hpf and become clearly apparent by 72 hpf^5^,^26^. To investigate why trabeculation occurs primarily in the ventricle and not the atrium^3^,^30^, we examined Nrg2a-mRFP expression at several stages of cardiac development. We used high-resolution confocal microscopy to image live *nrg2a*^*+/−*^ animals in the *Tg(kdrl:NLS-EGFP)* background, in which the endothelial cell nuclei are labelled with GFP^36^, at 52, 78 and 120 hpf (Fig. 2a–i). Nrg2a-mRFP expression appears in the endocardium at 52 hpf, particularly in the outer curvature of the ventricle (Fig. 2a–c, Supplementary Fig. 2a–c and Fig. 3a–c). Subsequently, Nrg2a-mRFP expression becomes stronger in ventricular and atrial endocardial cells (Fig. 2d–i, Supplementary Fig. 2d–i, Fig. 3d–i), although it is weaker in the atrioventricular (AV) canal (Fig. 2d–i and Supplementary Fig. 4a–c). Accordingly, *nrg2a* mutants develop functional AV valve leaflets (Supplementary Fig. 4d–i) as was observed in *erbb2* mutants^5^, suggesting that Nrg2a/Erbb2 signalling is not required for AV valve development.

Utilizing the Zeiss Efficient Navigation (ZEN) software, we measured the levels of Nrg2a-mRFP expression as well as the number of Nrg2a-mRFP positive endocardial cells at 78 hpf (Fig. 2j,k). These analyses show that ventricular endocardial cells express higher levels of Nrg2a-mRFP than atrial or AV canal endocardial cells (Fig. 2j), and that there are more Nrg2a-mRFP positive endocardial cells in the ventricle as compared to the other cardiac regions (Fig. 2k). We also found that on average (*N*=5 hearts), 97% and 90% of endocardial cells in the ventricle and atrium were positive for Nrg2a-mRFP expression, respectively (Supplementary Fig. 2j). Thus, ventricular cardiomyocytes might be exposed to a higher level of Nrg2a ligands than atrial ones, although atrial endocardial cells are clearly positive for Nrg2a-mRFP expression.

To examine whether the Nrg2a protein-trap expression in the endocardium accurately recapitulates endogenous *nrg2a* expression^34^,^35^, we carried out *in situ* hybridizations on 78 hpf hearts. We observed that *nrg2a* is more highly expressed in the ventricle, compared to the atrium, and that it is mainly restricted to the outer curvature (Supplementary Fig. 2k). This expression pattern is consistent with that of Nrg2a-mRFP.

### The cardiac jelly and the onset of trabeculation

The cardiac jelly is known to be pivotal in endo-myocardial interactions required for cardiac morphogenesis^4^,^37^, and its reduction during development has previously been reported in chicken and mice^4^,^38^. Using high-resolution confocal microscopy, we decided to investigate more closely the dynamics of cardiac jelly reduction around the onset of trabeculation in *Tg(kdrl:Hsa.HRAS-mCherry)*^39^*;Tg(myl7:EGFP-Hsa.HRAS)*^40^ animals in which endocardial and myocardial membranes are labelled in red and green, respectively. We found that at 48 hpf the cardiac jelly fully separates the endocardial and myocardial layers (Fig. 3a), and that its thickness gradually decreases in both chambers but more clearly in the ventricle (Fig. 3b–h). The timing of this decrease correlates with the appearance of the first trabeculae in the outer curvature of the ventricle by 60 hpf (Fig. 3b,c, arrowhead). By 96 hpf, the cardiac jelly appears to be fully gone in the ventricle while it remains in the atrium for at least the first 6 days of development (Fig. 3d–h).

To investigate whether Nrg2a signalling modulates the thickness of the cardiac jelly, we incrossed *nrg2a*^*+/−*^ animals in the *Tg(kdrl:Hsa.HRAS-mCherry);Tg(myl7:LIFEACT-GFP)* background, and found no obvious differences between wild types and mutants (Supplementary Fig. 5a–h), similar to what we observed in *nrg1*^*bns101*^ mutants and Erbb2 inhibitor-treated animals (Supplementary Fig. 5i–k), suggesting that Nrg/Erbb2 signalling is not involved in this process in zebrafish.

Altogether, these data show that the reduction of the cardiac jelly takes place in the ventricle as trabeculation starts, suggesting that it may determine the onset of this process, possibly by constituting a diffusion barrier for Nrg ligands.

### nrg2a overexpression in cardiomyocytes

**Figure 4:**
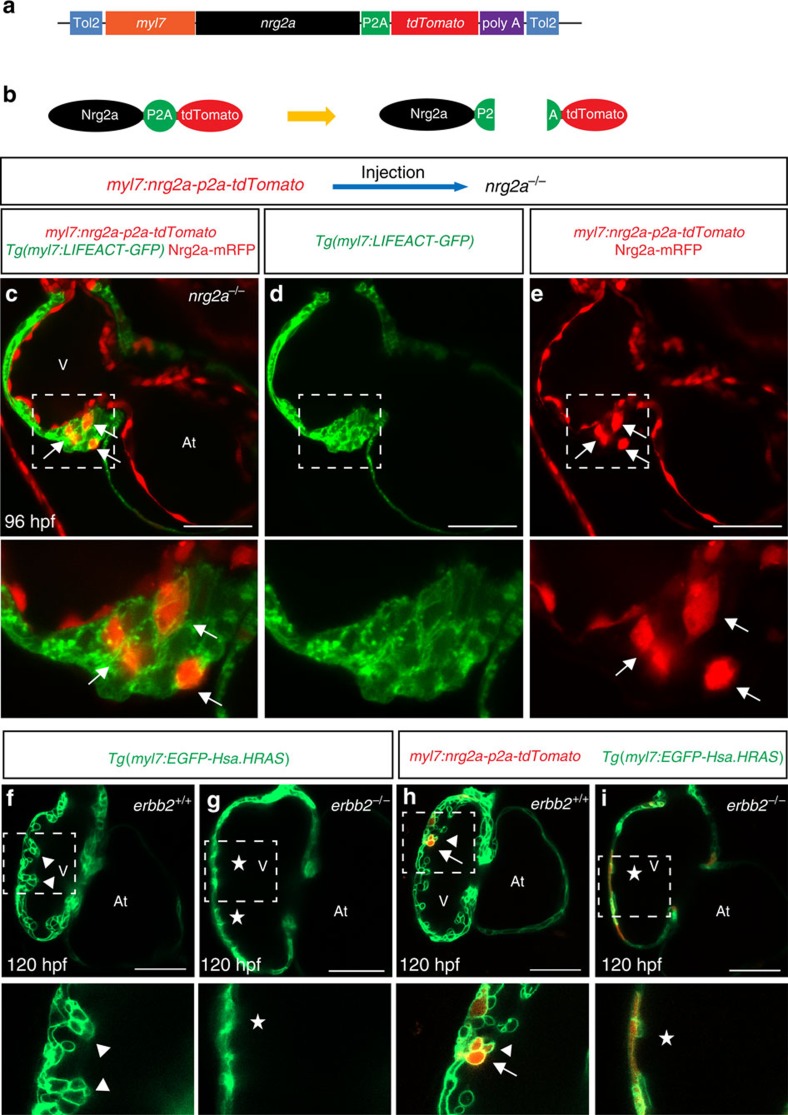
Myocardial nrg*2a* overexpression induces cardiomyocyte multilayering in *nrg2a* mutants. (**a**) Cartoon of myocardial specific *nrg2a* construct. (**b**) Schematic representation of Nrg2a protein tagged by tdTomato. Due to the presence of the P2A peptide, cleavage occurs right after protein translation to separate the Nrg2a from the tdTomato fluorescent protein. (**c**–**e**) 2D confocal images (mid-sagittal sections) of *Tg(myl7:LIFEACT-GFP);nrg2a*^*−/−*^ hearts injected with myocardial specific *nrg2a* construct (*myl7:nrg2a-p2a-tdTomato*) at the one-cell stage. Mosaic overexpression of *nrg2a* in *nrg2a*^*−/−*^ cardiomyocytes led to the formation of a multilayered myocardial wall which is outlined by a white dashed box and magnified (**c**–**e**); arrows point to *nrg2a* overexpressing cardiomyocytes. (**f**–**i**) Confocal images (mid-sagittal sections) of 120 hpf *Tg(myl7:EGFP-Hsa.HRAS)* hearts from *erbb2*^*+/−*^ incrosses injected with the *myl7:nrg2a-p2a-tdTomato* construct (**h**–**i**). Magnified images of dashed boxes are shown below **c**–**i**; arrows point to *nrg2a* overexpressing cardiomyocytes, arrowheads point to trabeculae and asterisks indicate lack of trabeculae. At, atrium; V, ventricle; scale bars, 50 μm.

The current model is that endocardial-derived Nrg ligands traverse the cardiac jelly to activate cardiomyocyte behaviour and proliferation^4^,^12^,^41^,^42^. To determine whether ectopic overexpression of *nrg2a* in cardiomyocytes enables them to form trabeculae in *nrg2a* mutants, we collected embryos from *Tg(myl7:LIFEACT-GFP)*;*nrg2a*^*+/−*^ incrosses and injected them with a *myl7:nrg2a-p2a-tdTomato* plasmid at the one-cell stage (Fig. 4a,b). This approach allows one to identify the *nrg2a* overexpressing cardiomyocytes by virtue of their expression of tdTomato. Confocal microscopy analysis showed that ectopic overexpression of *nrg2a* in *nrg2a* mutant ventricular cardiomyocytes could drive them to form a multilayered wall in most tdTomato positive hearts (13<15) (Fig. 4c–e). To determine whether Nrg2a signals via Erbb2 in cardiomyocytes, we ectopically overexpressed *nrg2a* in *erbb2*^*st61*^ mutant cardiomyocytes. Our results show that mosaic myocardial *nrg2a* overexpression did not rescue the trabeculation defects in *erbb2* mutants (Fig. 4f–i), indicating that Nrg2a must signal through Erbb2 in cardiomyocytes.

We also generated a stable myocardial-specific Nrg2a transgenic line by injecting wild-type embryos with the *myl7:nrg2a-p2a-tdTomato* plasmid. Although a majority of the injected animals survived to adulthood, some exhibited pericardial edema due to the presence of a much enlarged heart as compared to those of non-injected animals, indicating that mosaic expression of *nrg2a* in cardiomyocytes leads to cardiomegaly (Supplementary Fig. 6a–d), as expected from data in mouse using a transgene encoding constitutively active Erbb2 (ref. 21) and in zebrafish using *nrg1* overexpression^22^. Overall, these data indicate that ventricular cardiomyocytes are capable to respond to the myocardial overexpression of *nrg2a*.

### Atrial cardiomyocytes can respond to Nrg2a signalling

**Figure 5:**
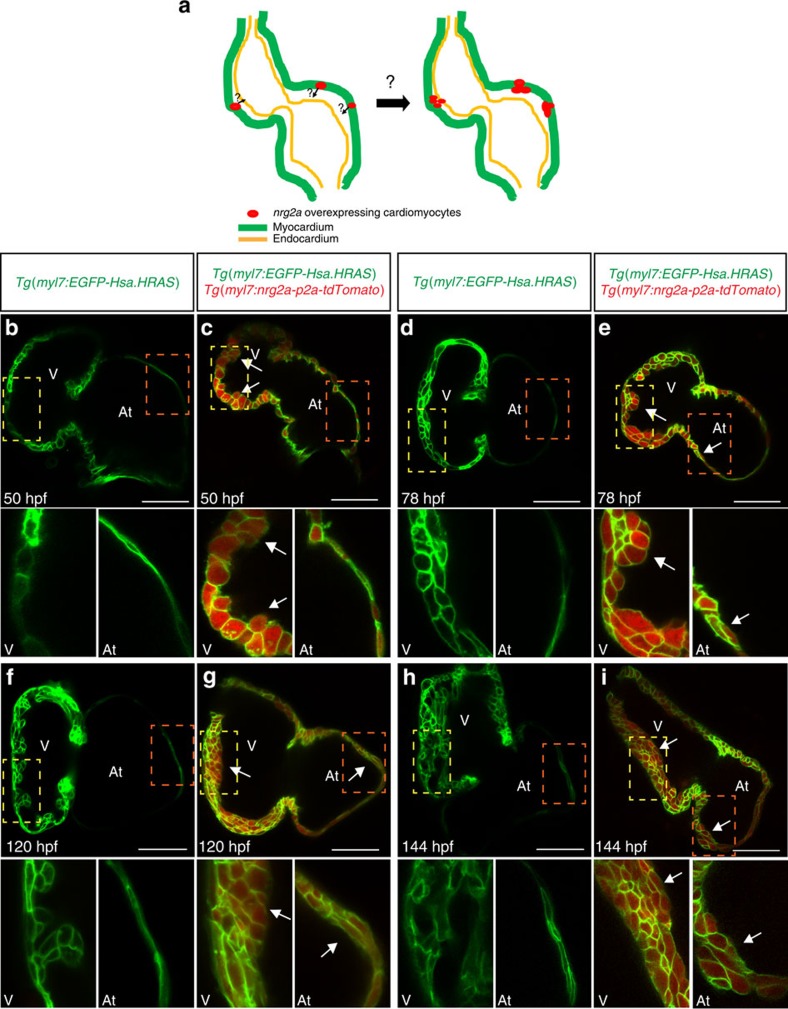
Myocardial specific *nrg2a* overexpression can lead atrial cardiomyocytes to form a multilayered wall. (**a**) Illustration of cardiomyocyte specific *nrg2a* overexpression in zebrafish. (**b**–**i**) 2D confocal images (mid-sagittal views) of *Tg(myl7:EGFP-Hsa.HRAS)* and *Tg(myl7:EGFP-Hsa.HRAS);Tg(myl7*:*nrg2a-p2a-tdTomato)* hearts at 50 (**b**,**c**), 78 (**d**,**e**), 120 (**f**,**g**) and 144  hpf (**h**,**i**); magnified images of dashed boxes are shown below each time point; arrows point to multilayered walls; At, atrium; V, ventricle; scale bars, 50 μm.

To further investigate the correlation between cardiac jelly thickness and the onset of trabeculation, we used our newly generated *Tg(myl7:nrg2a-p2a-tdTomato)* line to evaluate the behaviour of cardiomyocytes in the presence or absence of Nrg2a signalling (Fig. 5a). The cardiac jelly is present for at least the first 6 days of development in the zebrafish atrium, physically separating the endocardial and myocardial walls (Fig. 3), and thus possibly preventing myocardial cells from receiving endocardial-derived Nrg2a molecules. A myocardial specific *nrg2a* overexpression approach allows one to circumvent the hypothetical barrier function of the cardiac jelly and test whether atrial cardiomyocytes are in fact competent to respond to Nrg signalling. To better visualize cardiomyocyte boundaries, *Tg(myl7:nrg2a-p2a-tdTomato)* fish were outcrossed to the myocardial specific membrane line *Tg(myl7:EGFP-Hsa.HRAS).* Compared to *Tg(myl7:nrg2a-p2a-tdTomato)* negative animals, we observed that *nrg2a* overexpression in cardiomyocytes pushed them to form a bilayered wall in the ventricle as well as the atrium starting at 46 and 78 hpf, respectively (Fig. 5b–e, Supplementary Fig. 7a,b). Subsequently, constitutively active Nrg2a signalling resulted in cardiomyocyte multilayering in both ventricular and atrial walls (Fig. 5f–i). However, this cardiomyocyte response was blocked by Erbb2 inhibitor treatment starting at 36 hpf (Supplementary Fig. 8a–d). By crossing the *Tg(myl7:nrg2a-p2a-tdTomato)* line with a Fucci line (*Tg(myl7:mVenus-gmnn))* which labels proliferating cardiomyocytes in green^43^, we observed that ectopic myocardial expression of *nrg2a* increased the number of proliferating cardiomyocytes (Supplementary Fig. 9a–f). Overall, these data indicate that both ventricular and atrial cardiomyocytes can respond to Nrg/Erbb2 signalling.

### Contractility regulates endocardial nrg2a expression

**Figure 6:**
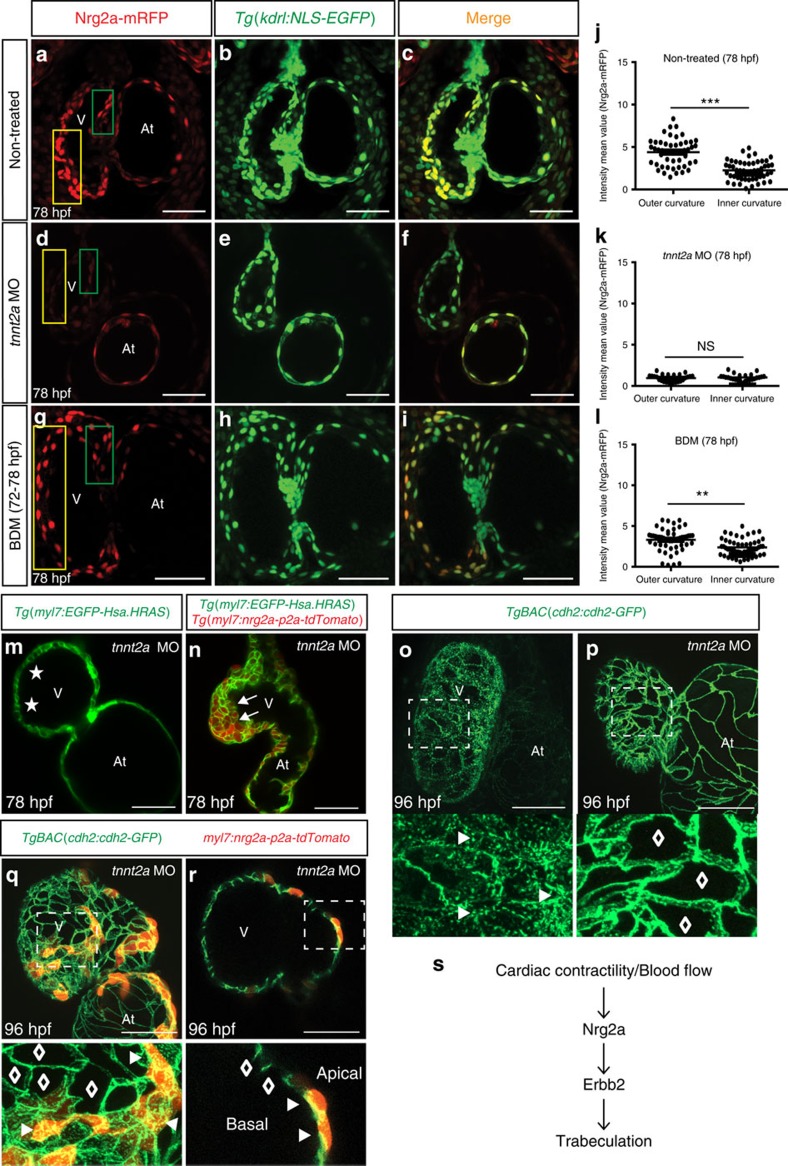
Contractility/blood flow is required for endocardial expression of *nrg2a* but not the ability of cardiomyocytes to respond to *nrg2a*. (**a**–**i**) Maximum intensity *z*-projections (25–30 *z*-stacks, mid-sagittal sections) of *Tg(kdrl:NLS-EGFP);nrg2a*^*+/−*^ hearts from non-treated (**a**–**c**), *tnnt2a* MO injected (**d**–**f**) and BDM treated (**g**–**i**) 78 hpf larvae; scale bars, 50 μm. (**j**–**l**) Graphs showing cell-based Nrg2a-mRFP intensity in outer curvature (yellow box) and inner curvature (green box) of hearts from non-treated (**j**), *tnnt2a* MO (**k**) and BDM treated (**l**) 78 hpf larvae; dots represent individual Nrg2a-mRFP expressing endocardial cells. Values represent means±s.e.m.; **P≤0.01, ***P≤0.001, NS (not significant), by Student's *t*-test. (**m,n**) 2D confocal images (mid-sagittal sections) of 78 hpf *Tg(myl7:EGFP-Hsa.HRAS)* (**m**) or *Tg(myl7:EGFP-Hsa.HRAS);Tg(myl7*:*nrg2a-p2a-tdTomato)* hearts (**n**) showing that myocardial overexpression of *nrg2a* can induce cardiomyocyte multilayering in *tnnt2a* morphants; asterisks and arrows indicate single-layered and multilayered ventricular walls, respectively. (**o**–**r**) Maximum intensity *z*-projections of *TgBAC(cdh2:cdh2-EGFP)* hearts from non-injected (**o**), injected with *tnnt2a* MO alone (**p**) or injected with *tnnt2a* MO and *myl7*:*nrg2a-p2a-tdTomato* plasmid (**q**,**r**) 96 hpf larvae. (**r**) 2D confocal image (sagittal section) of heart shown in **q**. Magnified images of dashed boxes are shown below (**o**–**r**); arrowheads and lozenges indicate presence and absence of Cdh2-EGFP proteins on the basal side of cardiomyocytes, respectively; At: atrium; V, ventricle; scale bars, 50 μm. (**s**) Schematic diagram of modulation of Nrg2a/Erbb2 signaling by cardiac contractility/blood flow.

Physical forces generated by contractility have been previously shown to modulate cardiac trabeculation^6^,^26^,^27^. Taking advantage of the *nrg2a* protein-trap line, we wanted to investigate how cardiac contractility/blood flow regulates *nrg2a* expression in the zebrafish heart. At 78 hpf, we observed that endocardial Nrg2a-mRFP is strongly expressed in the outer curvature of wild-type ventricles, where trabeculae are present, while it is clearly weaker in other endocardial cells (Fig. 6a–c,j). The levels of Nrg2a-mRFP expression in the heart were strongly reduced in the absence of cardiac contractility in *tnnt2a* morphants^44^ (Fig. 6d–f,k) and 2,3-butanedione monoxime (BDM)-treated larvae (Fig. 6g–i,l), indicating that contraction and/or blood flow is required for cardiac expression of Nrg2a-mRFP. To investigate whether there is also a role for cardiac contractility in regulating Erbb2 downstream signalling pathways, we injected *Tg(myl7:EGFP-Has.HRAS);Tg(myl7:nrg2a-202-p2a-tdTomato)* embryos with *tnnt2a* morpholinos and imaged them by confocal microscopy at 78 hpf. Notably, we observed that myocardial overexpression of *nrg2a* resulted in cardiomyocyte multilayering even in the absence of contractility (Fig. 6m,n). However, overexpressing *nrg2a* throughout the endothelium, including the endocardium, by using a stable *Tg(fli1a:nrg2a-p2a-tdtomato)* line did not cause a cardiomyocyte multilayering phenotype in *tnnt2a* morphants (Supplementary Fig. 10). By injecting low amount of *myl7:nrg2a-p2a-tdTomato* plasmid DNA in *tnnt2a* morphants, we also observed that mosaic expression of *nrg2a* recruits both *nrg2a* expressing and non-expressing cardiomyocytes to form a multilayered wall suggesting that *nrg2a* affects the behaviour of cardiomyocytes via both autocrine and paracrine signals (Supplementary Fig. 11). Furthermore, it has been recently reported that N-cadherin relocalizes to the basal side of cardiomyocytes during trabeculation^45^. We observed that *nrg2a* overexpression in non-contractile cardiomyocytes was sufficient to drive the relocalization of Cdh2-EGFP molecules to their basal side (Fig. 6o–r, Supplementary Movie 1) while they remained mostly localized on the lateral sides of non-*nrg2a* expressing cardiomyocytes, further indicating that cardiac contraction is not necessary for cardiomyocytes to respond to Nrg2a signalling. Altogether, these results support a model in which contractility/blood flow is required for endocardial expression of *nrg2a* but not the expression or function of Nrg2a/Erbb2 signalling effectors (Fig. 6s).

## Discussion

During trabeculation, cardiomyocytes undergo several morphogenetic changes required for the luminal growth and maintenance of the myocardial wall^2^,^5^,^6^,^8^,^33^,^43^,^45^. Taking advantage of high-resolution live imaging in the translucent zebrafish larvae, we investigated, using loss- and gain-of-function approaches, why trabeculation takes place primarily in the ventricle and how it is controlled by cardiac contractility/blood flow.

Given the importance of Nrg1 in cardiac trabeculation in mouse^11^,^46^,^47^, we investigated the role of Nrg1 in zebrafish and found that it is not required for trabeculation, a finding consistent with a very recent publication^48^. Instead we found that Nrg2a is required for trabeculation in zebrafish while loss of Nrg2 does not lead to any obvious cardiac defects in mouse^49^. Nrg signalling plays many essential roles in vertebrate development and homeostasis^5^,^12^,^41^,^50^,^51^, and it will be interesting to investigate ligand selection across evolutionary time. We also observed that Nrg1 and Nrg2a signalling are not required for AV valve formation in zebrafish, consistent with previous observations of *erbb2* mutants^5^. In mouse, Erbb2–Erbb3 heterodimer activity is required for early AV valve formation^52^,^53^, and it will be interesting to investigate the role of Erbb3 in zebrafish heart development.

To investigate why trabeculae form only in the ventricle, we used the *nrg2a*^*mn0237Gt/+*^ protein-trap line^35^ to monitor the endogenous expression of *nrg2a*, and found that it is expressed in the endocardium similar to mouse *Nrg1* and *Nrg2* (refs 54, 55). Our results also indicate that Nrg2a-mRFP is strongly expressed in endocardial cells lining the outer curvature of the ventricle whereas it is weaker in other endocardial cells suggesting that some ventricular cardiomyocytes might be exposed to higher level of Nrg2a signalling. One caveat here of course is that we examined the expression of a fusion protein which is likely to have very different stability than the endogenous Nrg2a protein.

Prior studies have also revealed the critical role of the cardiac jelly in endo-myocardial interactions^16^,^56^,^57^. Investigating the dynamics of cardiac jelly degradation during early cardiac development, we observed that by the time trabeculae appear, little cardiac jelly is left in the ventricle. By ectopic myocardial overexpression of *nrg2a*, we found that atrial cardiomyocytes could also respond to Nrg2a/Erbb2 signalling. We speculate that atrial cardiomyocytes receive less Nrg ligands, which are expressed by endocardial cells, due to the thickness of the cardiac jelly in the atrium and consequently do not form a multilayered wall.

When we overexpressed *nrg2a* in cardiomyocytes using the *myl7* promoter, we observed the formation of multiple continuous layers rather than the formation of trabeculae, possibly because of the high levels, even distribution and premature expression of *nrg2a*. We hypothesize that the expression of lower levels of *nrg2a* in single cardiomyocytes starting at the right stage would lead them to delaminate and seed trabeculae as observed in wild-type embryos, and new transgenic tools will be required to test this hypothesis. In addition, higher levels of Nrg2a signalling might drive cardiomyocyte proliferation.

Several groups have shown the importance of physical forces in cardiac morphogenesis^6^,^24^,^27^,^58^,^59^. It has recently been reported that in zebrafish cardiac contraction is required for cardiac *nrg1* expression but not for cardiac *erbb2* expression^27^. Here, we showed that contractility/blood flow is essential for cardiac expression of *nrg2a*. Interestingly, although *nrg1* upregulation by broad expression of a Notch intracellular domain was not sufficient to rescue trabeculation defects in *tnnt2a* morphants^27^, we could induce cardiomyocyte multilayering in these animals by myocardial overexpression of *nrg2a*. Moreover, we also showed that ectopic overexpression of *nrg2a* in non-contractile cardiomyocytes led to the relocalization of Cdh2-EGFP to their basal side. Overall, these data indicate that contractility/blood flow is indispensable for cardiac expression of *nrg2a* but not the ability of cardiomyocytes to respond to *nrg2a*.

We also observed that unlike myocardial specific *nrg2a* overexpression, endocardial specific *nrg2a* overexpression did not induce cardiomyocyte multilayering in *tnnt2a* morphants, likely due to the presence of a substantial gap between the endocardial and myocardial layers. These data further suggest that the thickness of the cardiac jelly may play an important role in determining when trabeculation starts.

In conclusion, our data indicate that trabeculae may initially form only in the ventricle in part because the cardiac jelly in the atrium does not get degraded as fast as it does in the ventricle. Atrial cardiomyocytes exhibit another kind of behaviour as they form the inner pectinate myofibers^60^,^61^, and it will be interesting to investigate the underlying signalling pathways. We also found that biomechanical forces work upstream of Nrg2a/Erbb2 signalling to modulate trabeculation in the zebrafish heart by modulating *nrg2a* expression in the endocardium. It will be important to identify the proteins involved in sensing these forces as well as the transcriptional effectors regulating *nrg2a* expression.

## Methods

### Zebrafish husbandry

Embryonic and adult zebrafish were raised and maintained under standard conditions^62^ and all animal experiments were done according to German Animal Protection Laws approved by the local governmental animal protection committee.

### Zebrafish transgenic and mutant lines

In this study, we used the following transgenic and mutant lines: *Tg(kdrl:Hsa.HRAS-mCherry)*^*s896*^ (ref. 39), *Tg(myl7:EGFP-Hsa.HRAS)*^*s883*^ (ref. 40), *Tg(myl7:LIFEACT-GFP)*^*s974*^ (ref. 33), *Tg(kdrl:NLS-EGFP)*^*ubs1*^ (ref. 36), *TgBAC(cdh2:cdh2-EGFP,crybb1:ECFP)*^*zf517*^ (ref. 63) abbreviated as *TgBAC(cdh2:cdh2-EGFP)*, *Tg(myl7:nrg2a202-p2a-tdTomato)*^*bns140*^ abbreviated as *Tg(myl7:nrg2a-p2a-tdTomato)*, *Tg(fli1a:nrg2a202-p2a-tdTomato)*^*bns199*^ abbreviated as *Tg(fli1a:nrg2a-p2a-tdTomato)* and *Tg(myl7:mVenus-gmnn)*^*ncv43Tg*^ (ref. 43) abbreviated as *Tg(myl7:mVenus-gmnn)*, *nrg2a*^*mn0237Gt*^ (ref. 35), *erbb2*^*st61*^ (ref. 64) and *nrg1*^*bns101*^.

### Generation of *nrg1* ^*bns101*^ mutant

*nrg1* mutants were generated by TALEN mutagenesis targeting exon 2. The following TALEN arms were constructed and assembled using the Golden Gate method^31^.

TALEN arm1: NG NG NG NN NN HD NI NN NN NI NI NI HD HD HD NI NN

TALEN arm2: HD NG NG NG NG HD HD NN NG NG HD NG NG NN NG NI HD HD

An amount of 100 pg of total TALEN RNA and 50 pg of GFP RNA (used to monitor injection efficiency) were coinjected into the cell at the one-cell stage.

The *nrg1*^*bns101*^allele was genotyped using high-resolution melt analysis (HRMA) with an Eco Real-Time PCR System (Illumina).

The following HRMA primers were used for PCR reactions:

*nrg1*-exon2- F1 5′-GAGATTTTGGCAGGAAACCC-3′

*nrg1*-exon2- R1 5′-CTTTATGCTTTTGGGTTTG-3′.

### Mosaic and stable overexpression of *nrg2a*

To generate a myocardial specific *nrg2a* overexpressing construct, the *nrg2a-202* coding sequence followed by *p2a-tdTomato* was cloned in a mini tol2 plasmid harbouring a *myl7* promoter using the Cold Fusion Cloning Kit (MC101A-1-SB). For mosaic expression of *nrg2a,* 15 pg of *myl7:nrg2a-202-p2a-tdTomato* plasmid DNA was coinjected with 10 ng of Tol2 RNA into the cell at the one-cell stage.

To establish the *Tg(myl7:nrg2a202-p2a-Tomato)*^*bns140*^ line, injected larvae (F0) were screened for myocardial tdTomato fluorescence and raised to adulthood. After identifying founders, F1 embryos positive for fluorescence in their cardiomyocyte were raised to adulthood.

### Morpholino injections

To stop cardiac contraction, 0.5 ng of a *tnnt2a* morpholino (5′-CATGTTTGCTCTGATCTGACACGCA-3′) was injected into one-cell stage embryos^44^.

### Chemical treatments

To prevent contractility for 6 h (short term treatment), 72 hpf zebrafish larvae were exposed to 20 mM BDM^65^. To block Erbb2 signalling, dechorionated embryos were treated with 10 μM of the Erbb2 inhibitor PD 168393 (Calbiochem) from 36 to 96 hpf.

### *In vivo* confocal imaging and data processing

Zebrafish embryos and larvae were mounted and anesthetized in 1.5% low-melt agarose (Sigma) containing 0.2 per cent tricaine on glass-bottom dishes. Before the gel solidified, the samples were manually oriented towards the microscope lens to enhance optical access to the heart. Images were captured with a Zeiss LSM780 or a spinning disk (CSU-X1 Yokogawa) confocal microscope using × 40 (1.1 NA) and × 40 (1.15 NA) water immersion lenses, respectively. The confocal data were then processed with the ZEN 2012 software (black edition). Nrg2a-mRFP intensity per cell was measured with the ZEN 2012 software (blue edition).

### Whole mount *in situ* hybridization

To perform whole mount *in situ* hybridization^66^, 78 hpf zebrafish larvae were fixed in 4% paraformaldehyde overnight at 4 °C and subsequently dehydrated in 100% methanol at −20 °C. Larvae were rehydrated with 1 × PBS and permeabilized by digestion with proteinase K (10 μg ml^−1^) at room temperature for 30 min. After washing with 1 × PBT (1 × phosphate buffered saline (PBS), 0.1% Tween 20 (vol/vol)), larvae were hybridized with 200 ng of *nrg2a* antisense DIG-labelled RNA probe overnight at 70 °C. The hybridized probes were then detected with alkaline phosphatase-conjugated anti-Digoxigenin antibody (Roche, dilution 1:1,000) for three hours at room temperature and the signal was visualized with BM purple (Roche).

The probe for *nrg2a* was amplified from 78 hpf heart cDNA using *nrg2a* forward 5′-TCTGTGTGGCTCTTTTGGTG-3′ and reverse 5′-GTGCGTCAGTCTGGTCTGTG-3′ primers. The PCR fragment was subcloned into pGEM-T.

### Statistical analysis

Data were processed with the Prism5 software. Values are presented as mean±s.e. *P* values (*P≤0.05, **P≤0.01, ***P≤0.001) were calculated using Student's *t*-test.

### Data availability

The authors declare that all data supporting the findings of this study are available within the article and its Supplementary Information files or from the corresponding author upon reasonable request.

## Additional information

**How to cite this article:** Rasouli, S. J. *et al*. Regulation of cardiomyocyte behavior in zebrafish trabeculation by Neuregulin 2a signaling. *Nat. Commun.* **8,** 15281 doi: 10.1038/ncomms15281 (2017).

**Publisher's note:** Springer Nature remains neutral with regard to jurisdictional claims in published maps and institutional affiliations.

## Supplementary Material

Supplementary Information Supplementary Figures, Supplementary References.

Supplementary Movie 1 Overexpression of nrg2a in non-contractile cardiomyocytes induces basal relocalization of Cdh2-EGFP. Movie reconstructed from 9 confocal sagittal sections (Z-stacks from apical to basal) of the Tg(myl7:EGFP-Hsa.HRAS) heart shown in Figure 6q showing the relocalization of Cdh2-EGFP proteins to the basal side of cardiomyocytes; arrows point to cardiomyocytes with basal Cdh2-EGFP molecules; scale bar, 50 μm.
