# Supplementary material for: Regulation of cardiomyocyte behavior in zebrafish trabeculation by Neuregulin 2a signaling
Source: Nat Commun. 2017 May 9;8:15281. doi: 10.1038/ncomms15281 (PMC5477525; doi:10.1038/ncomms15281)
Supplement: Supplementary Information — Supplementary Figures, Supplementary References. [file ncomms15281-s1.pdf]

Supplementary Figures

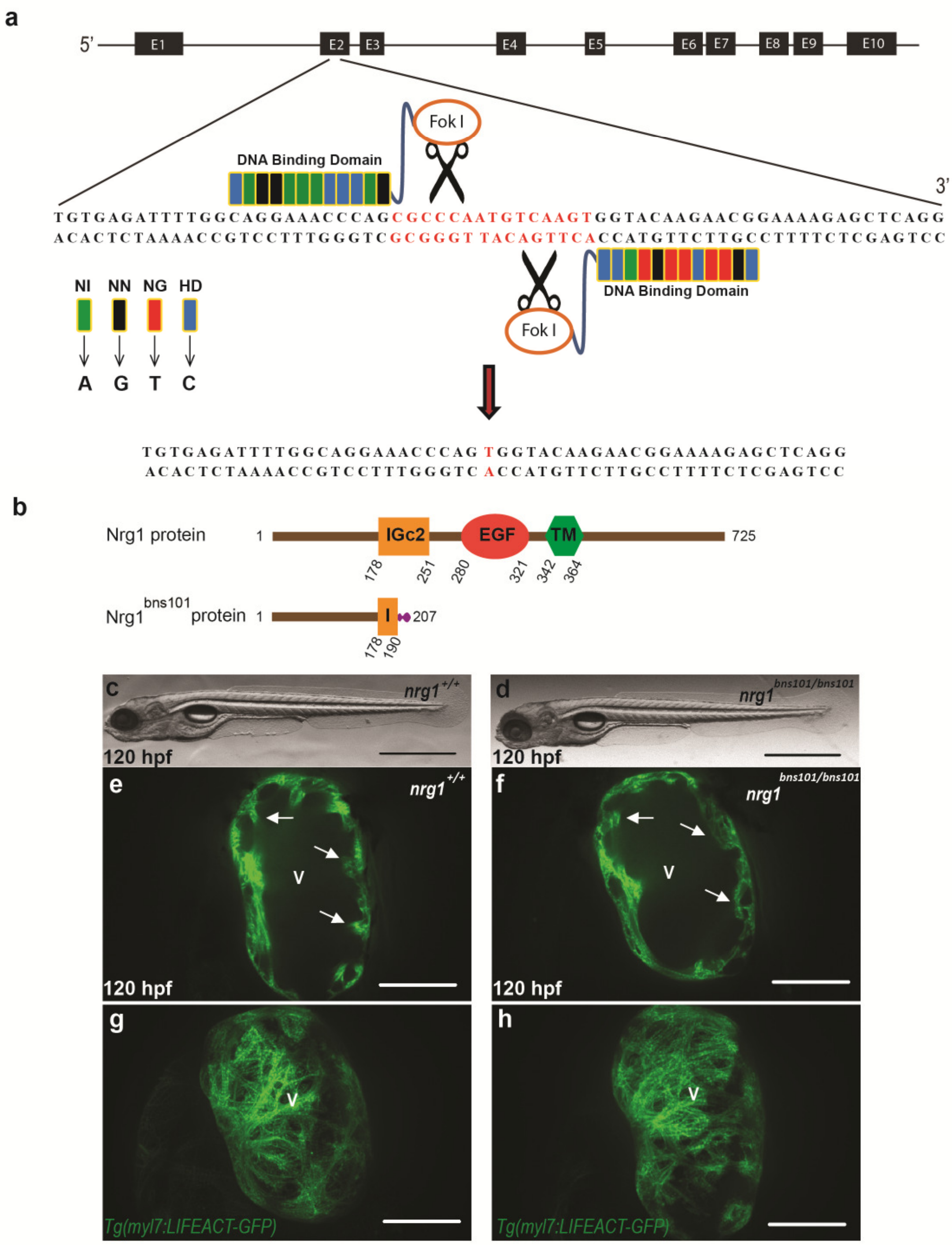

Supplementary Figure 1. ***nrg1$^{bns101/bns101}$*** **embryos develop a functional heart and survive to adulthood**

(a-b) Cartoon of Talen-induced *nrg1* mutation with a 14-base-pair deletion in exon 2 **(a)**; this deletion causes a frame shift leading to a premature stop codon in exon 2 resulting in a

predicted truncated protein **(b).** Notably, the predicted truncated Nrg1 protein in *nrg1*$^{bns101/bns101}$ animals lacks the Ig-like, EGF-like and TM domains that have all been reported to be essential for mouse cardiac trabeculation [1, 2, 3]. If the first ATG downstream of the 14 bp deletion is used to reinitiate translation, a protein consisting of part of the TM domain and the carboxy terminal domain will be made. However, the type IIIa Nrg1 isoform [4] is not predicted to be affected by the *bns101* mutation. **(c-d)** *nrg1*$^{bns101/bns101}$ larvae do not exhibit any gross morphological defects as compared to wild-type; lateral views, anterior to the left; scale bars, 0.5 mm. **(e-h)** Trabeculation appears unaffected in *nrg1*^{bns101} mutants. 120 hpf larvae from *Tg(myl7:LIFEACT-GFP); nrg1*$^{bns101/+}$ incrosses imaged by spinning disk confocal microscopy; 2D views (mid-sagittal sections) **(e and f),** and 3D maximum intensity z-projections **(g and h)**; V: ventricle; arrows point to trabecular cardiomyocytes; scale bars, 50 µm.

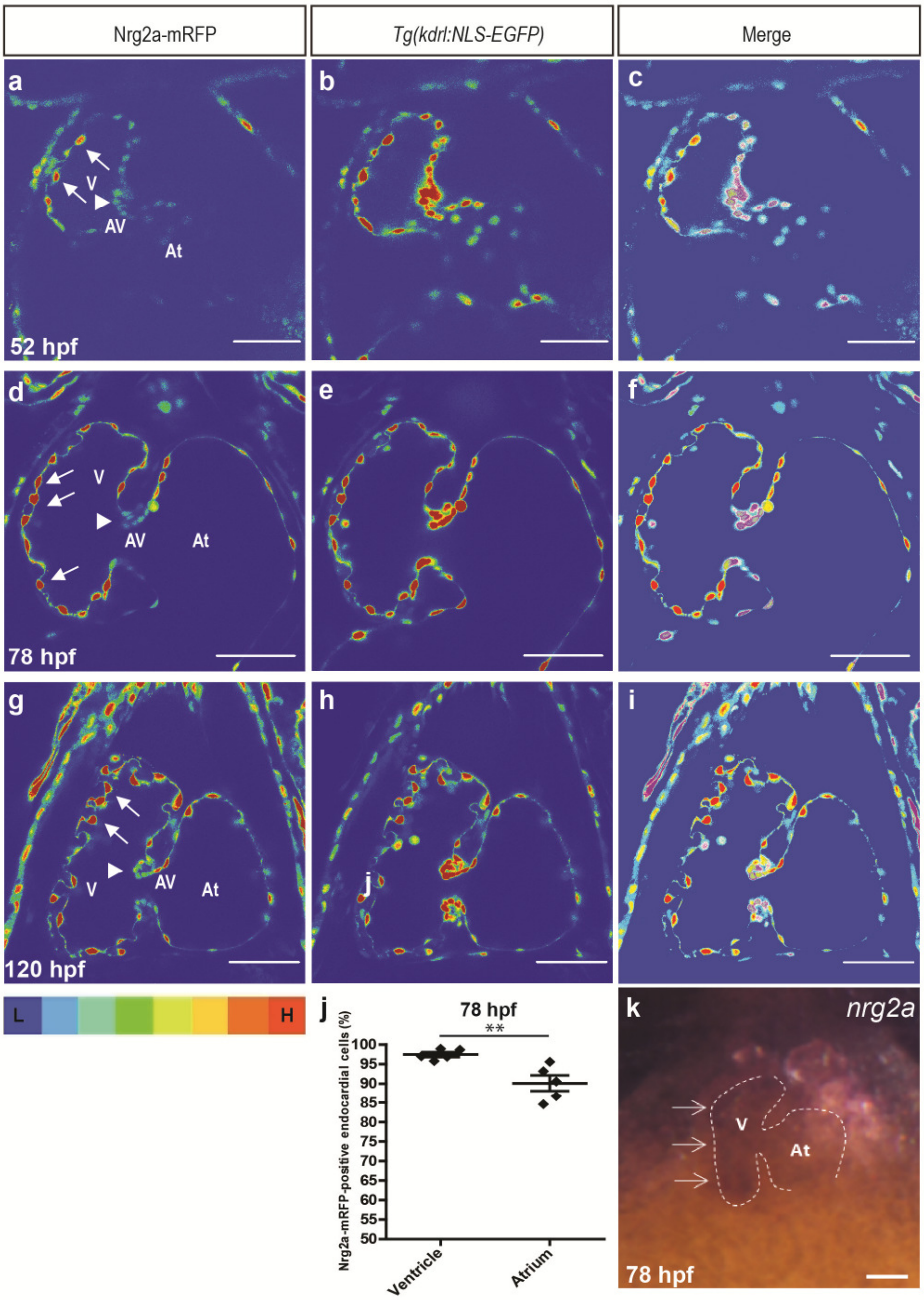

Supplementary Figure 2. Cardiac Nrg2a-mRFP expression during embryonic and larval development

(a-i) Mid-sagittal confocal images of *Tg(kdrl:NLS-EGFP)*;*nrg2a*$^{+/-}$ hearts; heat map; low intensity (blue) to high intensity(red) at 52 **(a-c)**, 78 **(d-f)** and 120 **(g-i)** hpf; arrows and arrowheads point to endocardial cells in ventricular outer curvature and superior AV valve leaflet, respectively. **(j)** Graph showing that on average there is a higher percentage of endocardial cells positive for Nrg2a-mRFP in the ventricle than in the atrium at 78 hpf; dots in this graph represent individual hearts; N=5 hearts; values represent means ± SEM; ** P≤ 0.01 by Student's *t*-test. **(k)** *In situ* hybridization for *nrg2a* expression in 78 hpf heart. V: ventricle, AV: atrioventricular canal, At: atrium; scale bars, 50 µm.

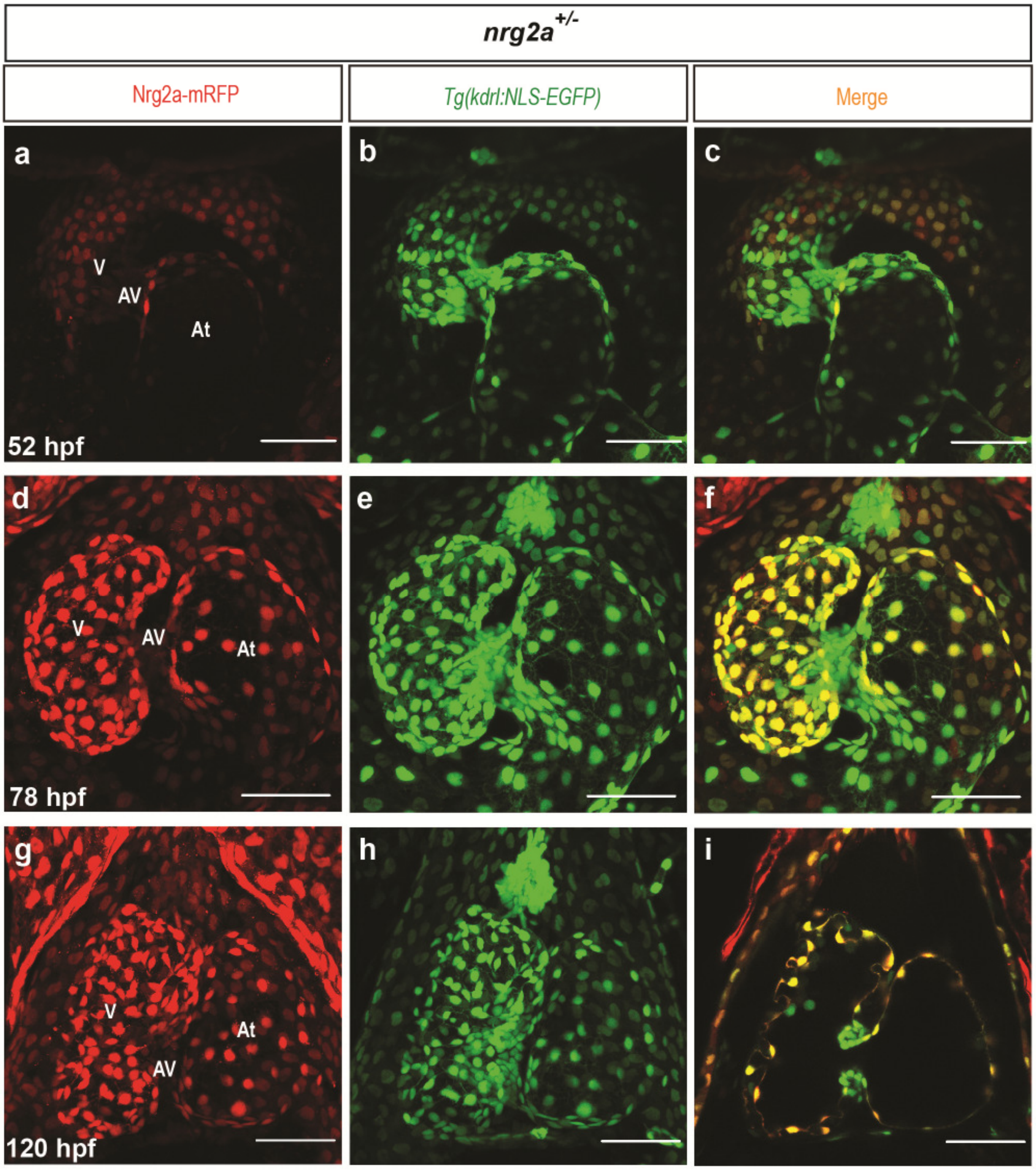

Supplementary Figure 3. Spatiotemporal pattern of cardiac Nrg2a-mRFP expression during embryonic and larval development

(a-i) 3D views (maximum intensity z-projections) of *Tg(kdrl:NLS-EGFP)*;*nrg2a*$^{+/-}$ hearts at 52 **(a-c)**, 78 **(d-f)** and 120 **(g-i)** hpf; Nrg2a-mRFP expression appears stronger in the ventricle than in the atrium or AV canal; V: ventricle, AV: atrioventricular canal, At: atrium; scale bars, 50 µm.

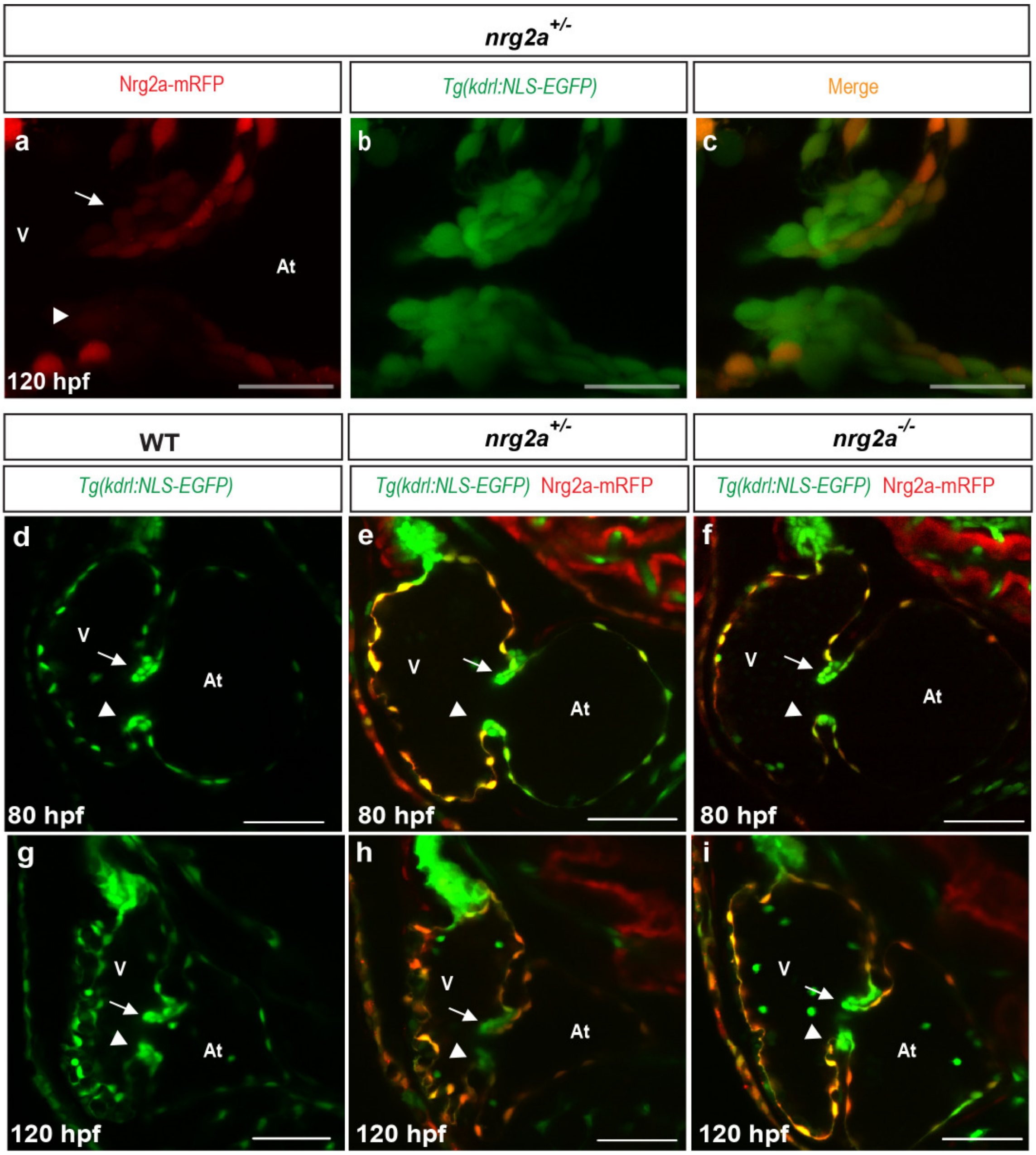

Supplementary Figure 4. ***nrg2a***$^{-/-}$ **hearts develop functional atrioventricular valves**

(a-c) Maximum intensity z-projections (25 z-stacks, sagittal sections) of 120 hpf *Tg(kdrl:NLS-EGFP)*;*nrg2a*$^{+/-}$ AV canal showing that Nrg2a-mRFP is weakly expressed in the AV valve leaflets. **(d-i)** Mid-sagittal confocal images of hearts from *Tg(kdrl:NLS-EGFP)*;*nrg2a*$^{+/-}$ incrosses at 80 **(d-f)** and 120 **(g-i)** hpf; arrows and arrowheads point to superior and inferior valve leaflets, respectively; V: ventricle, AV: atrioventricular canal, At: atrium; scale bars, 50 µm.

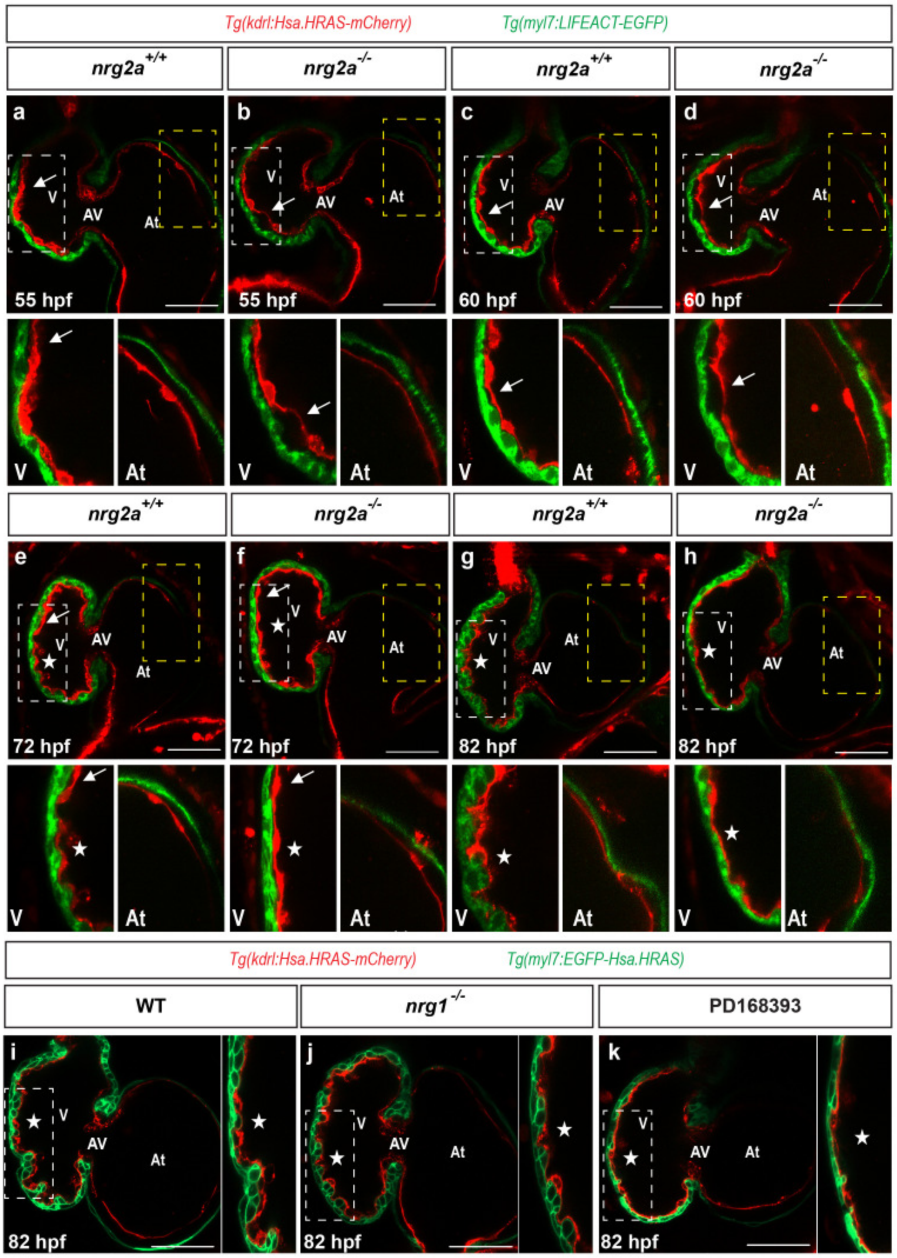

Supplementary Figure 5. Developmental analysis of cardiac jelly thickness in the presence or absence of Nrg/Erbb2 signaling

(a-h) Mid-sagittal views of wild-type and *nrg2a*$^{-/-}$ hearts from *Tg(kdrl:Hsa.HRAS-mCherry);Tg(myl7:LIFEACT-GFP);nrg2a*$^{+/-}$ incrosses at 55 **(a-b)**, 60 **(c-d)**, 72 **(e-f)** and 80 **(g-h)** hpf; the myocardium and endocardium are labeled in green and red, respectively; magnified images of dashed boxes are shown below each time point. The thickness of the cardiac jelly progressively diminishes in wild-type and *nrg2a*$^{-/-}$ hearts. **(i-k)** Confocal sagittal sections of 82 hpf *Tg(kdrl:Hsa.HRAS-mCherry);Tg(myl7:EGFP-Hsa.HRAS)* hearts from wild-type **(i)**, *nrg1*$^{-/-}$ mutant **(j)** or Erbb2 inhibitor (PD168363) treated animals showing that cardiac jelly reduction also appears to be an Nrg1/Erbb2 independent process; magnified views of dashed boxes in **i-h** are shown on the right; arrows and asterisks indicate the presence and absence of cardiac jelly, respectively; V: ventricle, AV: atrioventricular canal, At: atrium; scale bars, 50 µm.

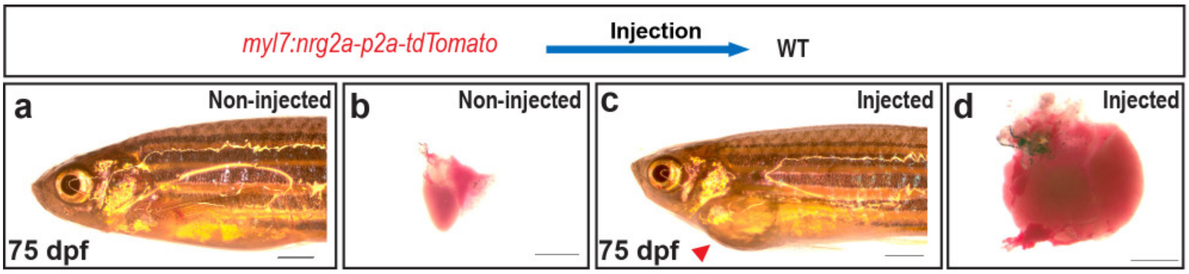

Supplementary Figure 6. Myocardial *nrg2a* overexpression causes cardiomegaly in wild-type fish

(a-d) A number of injected F0 wild-type fish (6 out of 31) exhibited pericardial edema at juvenile and adult stages as compared to non-injected ones; lateral views of non-injected **(a)** and injected **(c)** zebrafish imaged by brightfield microscopy at 75 dpf; red arrowhead in **c** points to pericardial edema; scale bars, 1mm. 75 dpf extracted hearts from non-injected **(b)** and injected **(d)** fish; scale bars, 200 µm.

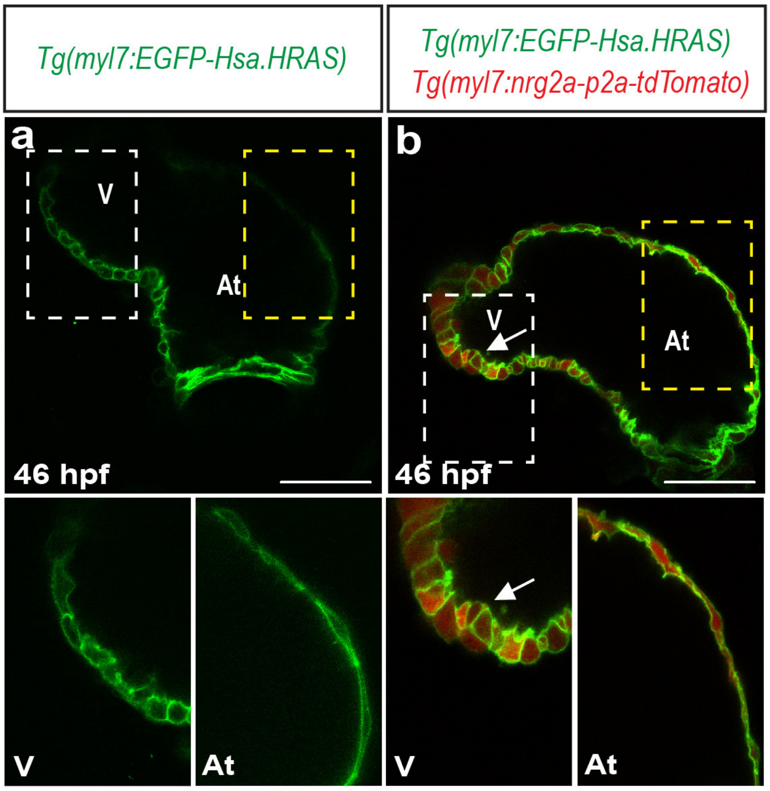

Supplementary Figure 7. Overexpression of *nrg2a* under control of a myocardial specific promoter induces cardiomyocyte bilayering in the ventricle by 46 hpf

(a-b) 2D ventral views (mid-sagittal sections) of 46 hpf *Tg(myl7:EGFP-Hsa.HRAS)* **(a)** or *Tg(myl7:EGFP-Hsa.HRAS);Tg(myl7:nrg2a-p2a-tdTomato)* **(b)** hearts; Magnified images of dashed boxes are shown below **a** and **b**; arrows point to bilayered wall; V: ventricle, At: atrium; scale bars, 50 µm.

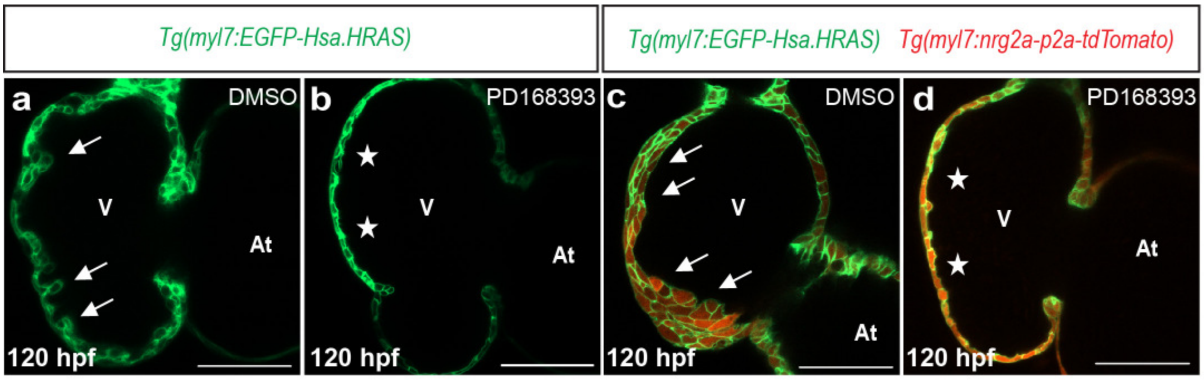

Supplementary Figure 8. Ectopic *nrg2a* overexpression in cardiomyocytes requires Erbb2 function

(a-d) Confocal sagittal sections of *Tg(myl7:EGFP-Hsa.HRAS)* **(a-b)** or *Tg(myl7:EGFP-Hsa.HRAS);Tg(myl7:nrg2a-p2a-tdTomato)* **(c-d)** hearts, treated with DMSO **(a and c)** or the Erbb2 inhibitor PD168363 **(b and d)** showing that *nrg2a* overexpression-induced cardiomyocyte multilayering requires Erbb2 function; asterisks and arrows indicate single-layered and multilayered ventricular walls, respectively; V: ventricle, At: atrium; scale bars, 50 µm.

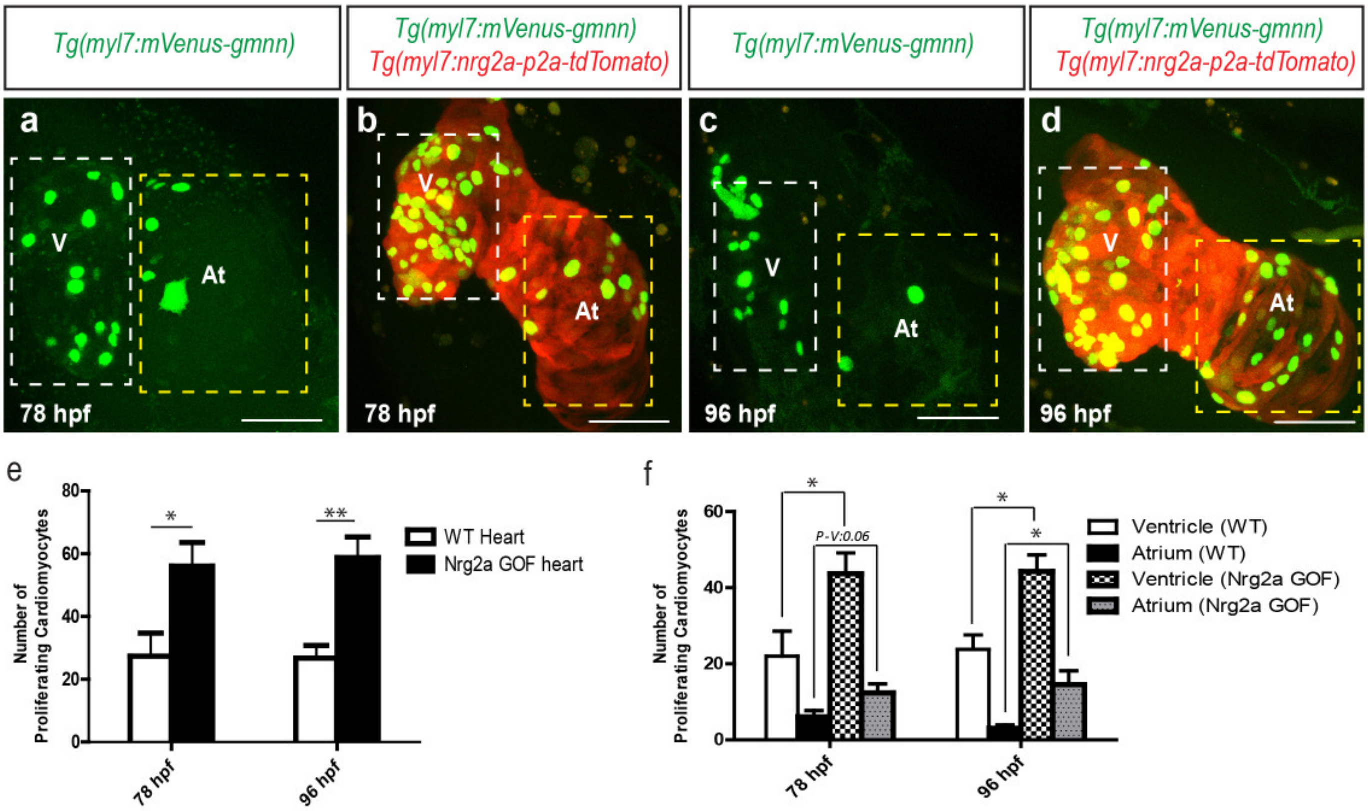

Supplementary Figure 9. Myocardial specific *nrg2a* overexpression leads to an increase in cardiomyocyte proliferation

(a-d) 3D views (maximum intensity z-projections) of *Tg(myl7:mVenus-gmnn)* **(a and c)** or *Tg(myl7:mVenus-gmnn);Tg(myl7:nrg2a-p2a-tdTomato)* **(b and d)** hearts at 78 **(a-b)** and 96 **(c-d)** hpf. Proliferating cardiomyocytes are labeled in green; white and yellow dashed boxes outline the ventricular and atrial chambers, respectively; V: ventricle, At: atrium; scale bars, 50 µm. **(e-f)** Graphs showing the average number of proliferating cardiomyocytes per heart **(e)** and per ventricular or atrial **(f)** chamber at 78 and 96 hpf; N=9 hearts; values represent means ± SEM; * P≤ 0.05,** P≤ 0.01 by Student's *t*-test.

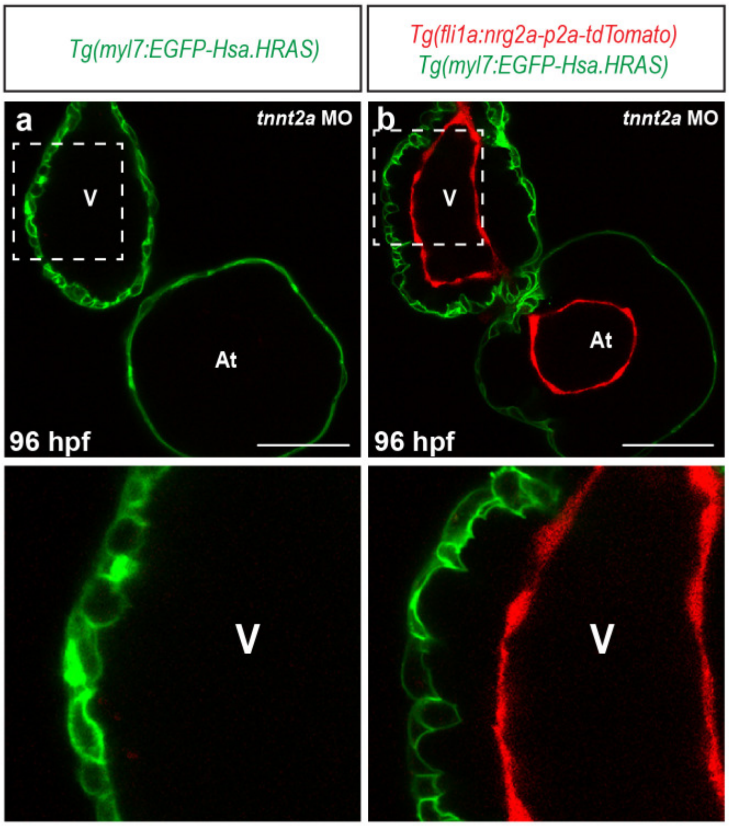

Supplementary Figure 10. Endocardial specific overexpression of *nrg2a* does not induce cardiomyocyte multilayering in *tnnt2a* morphants

(a-b) 2D ventral views (mid-sagittal sections) of 96 hpf *Tg(myl7:EGFP-Hsa.HRAS)* **(a)** or *Tg(fli1a:nrg2a-p2a-tdTomato);Tg(myl7:EGFP-Hsa.HRAS)* **(b)** hearts from *tnnt2a* MO (0.5 ng) injected animals; magnified images of dashed boxes are shown below **a** and **b**; myocardium and endocardium are labeled in green and red, respectively; V: ventricle, At: atrium; scale bars, 50 µm.

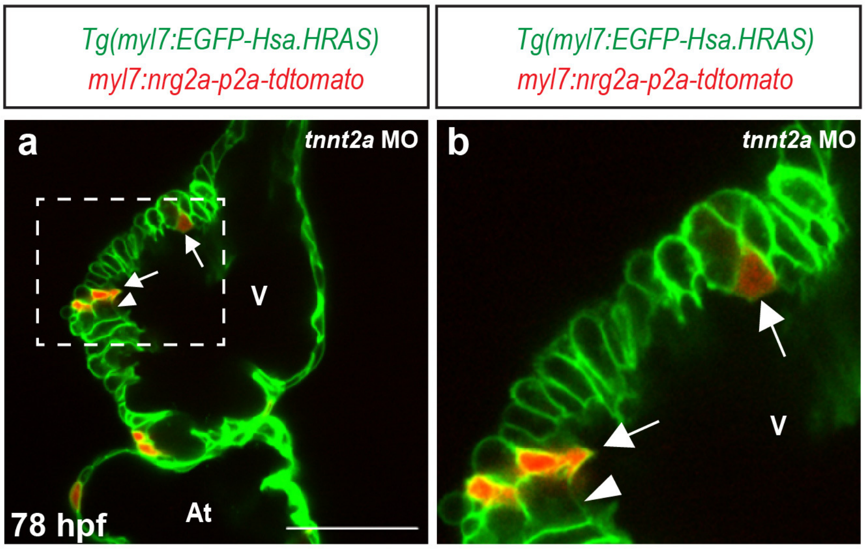

Supplementary Figure 11. Nrg2a can affect cardiomyocyte behavior via both autocrine and paracrine signals

(a-b) 2D ventral views (mid-sagittal sections) of 78 hpf *Tg(myl7:EGFP-Hsa.HRAS)* hearts from animals injected with *tnnt2a* MO (0.5 ng) and *myl7:nrg2a-p2a-tdTomato* DNA (5-10 pg). This low amount of DNA allows one to get single cardiomyocytes to overexpress the *nrg2a* transgene; arrows and arrowhead point to *nrg2a-p2a-tdTomato* expressing and non-expressing cardiomyocytes, respectively; higher magnification of dashed box in **a** is shown in **b**; V: ventricle, At: atrium; scale bar, 50 µm.

Supplementary References

1. Erickson SL*, et al.* ErbB3 is required for normal cerebellar and cardiac development: a comparison with ErbB2-and heregulin-deficient mice. *Development* **124**, 4999-5011 (1997).

2. Kramer R, Bucay N, Kane DJ, Martin LE, Tarpley JE, Theill LE. Neuregulins with an Ig-like domain are essential for mouse myocardial and neuronal development. *Proceedings of the National Academy of Sciences* **93**, 4833-4838 (1996).

3. Lai D*, et al.* Neuregulin 1 sustains the gene regulatory network in both trabecular and nontrabecular myocardium. *Circulation research* **107**, 715-727 (2010).

4. Honjo Y, Kniss J, Eisen JS. Neuregulin-mediated ErbB3 signaling is required for formation of zebrafish dorsal root ganglion neurons. *Development* **135**, 2615-2625 (2008).
